# The Effect of Workplace Violence on the Health of Healthcare Workers: Empirical Evidence From a Multicenter Cross-Sectional Study in China

**DOI:** 10.3389/ijph.2025.1608523

**Published:** 2025-10-27

**Authors:** Tao Luo, Xiumei Tang, Li Ma, Weimin Li

**Affiliations:** ^1^ Business School, Sichuan University, Chengdu, China; ^2^ Health Management Center, General Practice Medical Center, West China Hospital, Sichuan University, Chengdu, China; ^3^ Institute of Hospital Management, West China Hospital, Sichuan University, Chengdu, China; ^4^ Department of Respiratory and Critical Care Medicine, West China Hospital, Sichuan University, Chengdu, China; ^5^ Outpatient Department, West China Hospital, Sichuan University, Chengdu, China; ^6^ West China School of Nursing, Sichuan University, Chengdu, China

**Keywords:** China, healthcare workers, instrumental variable, propensity score matching, workplace violence

## Abstract

**Objectives:**

To investigate the causal relationship between workplace violence and health outcomes among healthcare workers, addressing gaps in evidence on its mechanisms and heterogeneous effects.

**Methods:**

A nationally representative cohort of 4,255 Chinese healthcare workers was surveyed via four-stage stratified sampling. Causal effects were estimated using multiple linear models and ordered logit model, with robustness checks via propensity score matching and instrumental variables to mitigate endogeneity.

**Results:**

Workplace violence reduces the probability of healthcare workers experiencing improved health by 12.9% (p = 0.000), with this effect persisting even after considering endogeneity. Physical violence had the most substantial impact, while psychological and verbal violence also contributed. Professional values mediated the effect. Vulnerable subgroups included women, younger workers, lower-ranking staff, and non-tertiary hospital employees.

**Conclusion:**

This study provides causal evidence that workplace violence undermines the health of healthcare workers, with implications for hospital policies and occupational safety standards. Interventions should prioritize physical violence prevention, support for high-risk groups, and value-based resilience training.

## Introduction

Healthcare workers (HCWs), numbering approximately 104 million globally, play a vital role in delivering care, yet their physical and psychological wellbeing has mainly been overlooked [1, 2]. They face numerous stressors, including heavy workloads, long shifts, high-paced environments, and exposure to physical and psychological risks, further intensified by moral conflicts, workplace bullying, lack of social support, and job insecurity [3, 4]. These challenges lead to mental health issues such as dissatisfaction, stress, depression, anxiety, sleep disorders, compassion fatigue, and burnout, with 1.0% of physicians reporting suicide attempts and 17% experiencing suicidal ideation [5, 6]. According to a recent meta-analysis of 253 studies involving 331,544 participants, 61.9% of HCWs have experienced some form of WPV [7]. The consequences of WPV are profound, negatively impacting both physical and mental health. Anxiety, depression, posttraumatic stress disorder (PTSD), and other psychological conditions can lead to reduced job satisfaction, lower professional performance, increased turnover, and higher burnout rates [8–11]. Moreover, treating and compensating employees injured by WPV incurs significant costs and prolonged absences, further straining healthcare systems [12–15].

Existing research primarily examines hospital-based WPV through surveys, including assessments of risk factors, investigation of incident rates, management approaches, and consequences of people who encountered WPV [16]. Many researches indicates that WPV was significantly associated with objective level factors (age, gender, education level, professional status, workload, and work experience) [17], organizational level factors (shift work, excessive service volume, and high-stress situations) [18, 19], and personal level factors (history of drug or alcohol abuse, violence, or psychiatric conditions) [18].

WPV manifests in a spectrum of detrimental health effects, mediated by complex behavioral pathways. In the study of Zhao et al. [20], depression plays a key mediating role between WPV and occupational burnout. Havaei et al. [21] reveal that burnout mediated the relationship between WPV and health outcomes. However, given the importance of HCWs’ health, research gaps still need to be urgently filled. First, current research has yet to rigorously and scientifically explore whether and to what extent WPV affects the health of HCWs. Although some studies have discussed the adverse effects of WPV, there is a lack of direct examination. Second, previous studies have not yet established robust causality, most of the studies did not consider the endogeneity of the estimates, which led to the fact that their studies perhaps only provided evidence of the correlation between WPV and health outcomes [22, 23], and their estimates may even have been biased. We know little about the underlying mechanisms by which WPV affects the health of HCWs, which hinders our in-depth understanding of the effects of WPV. Professional value refers to the perceived value of their work, which affects workers’ productivity and job satisfaction [11]. Recent studies have shown that professional values are a key factor influencing the health of farmers or workers [24, 25]. However, given the distinct nature of healthcare work, the mediating effect of professional value among HCWs remains unexplored and requires further investigation.

Building on the current research gaps, this study leverages a large-scale dataset of Chinese HCWs to examine the effects of WPV on their health scientifically. It aims to evaluate the overall impact of WPV, identify the type that poses the most significant harm, and investigate how its effects vary based on the hospitals’ and HCWs’ characteristics. Additionally, the study seeks to deepen understanding by exploring professional value as a mediating factor. The findings aim to inform policy development to address health-related challenges in this field.

## Methods

### Data Source

This is a multicenter cross-sectional study conducted between October 2022 and March 2023 in China and using a four-stage stratified sampling technique [11]. With approval from the administrations of each hospital, email invitations were sent to the HCWs. Participants were required to provide written informed consent before accessing the questionnaire: before accessing the survey questionnaires, written informed consent was provided, and they were assured of their anonymity, informed that participation was voluntary, and had the option to withdraw from the study at any time without consequence. After consent was given, participants were given access to the questionnaire, which was designed to take approximately 15 min to complete, based on a pilot trial with healthcare workers who were not involved in the main study. All procedures performed in studies involving human participants were in accordance with the ethical standards of the institutional and/or national research committee (Ethics Committee of West China Hospital, No. 2023822) and with the 1964 Helsinki declaration and its later amendments or comparable ethical standards [26].

### Variable Definition

This study’s key dependent variable is HCWs’ health status, measured using self-rated health (SRH) [24]. SRH is a widely utilized method for assessing health due to its simplicity, affordability, and self-reported nature [27]. For this study, participants assessed their health status by responding to the question, “How is your health status?” using a 5-point scale: 1 for “Very Unhealthy,” 2 for “Unhealthy,” 3 for “Average,” 4 for “Healthy,” and 5 for “Very Healthy.” This approach is particularly valuable in large population surveys, serving as a practical starting point for discussions about individuals’ health perceptions. Poor SRH has been shown to independently predict future health outcomes, such as disability, mortality, physical dysfunction, cardiovascular disease, and increased healthcare utilization [28–30]. SRH is strongly correlated with various biomarkers and is recognized as a reliable predictor of mortality, even when controlling for other health indicators [31]. Its predictive strength lies in its ability to reflect physical and mental health, providing a holistic view of an individual’s wellbeing. Moreover, SRH can detect subtle bodily changes that conventional empirical studies may overlook, highlighting its significance in understanding and predicting health outcomes through the interplay of social and biological mechanisms [24, 32].

The core explanatory variable in this study is WPV. The Chinese version of the Workplace Violence Scale was used [33]. This scale has been validated for its reliability and accuracy (with a Cronbach’s alpha of 0.92) in the Chinese healthcare context. It includes five categories of violence: PA, EA, T, VSH, and SA. Respondents rated their exposure to each type of violence on a scale from 0 to 3, with 0 representing no incidents, 1 for one incident, 2 for two or three incidents, and 3 for more than three incidents in the past year. The total score ranges from 0 to 15 and is the sum of all five item scores. The survey included clear definitions for each type of violence. Further details about the questionnaire are available in Additional file 1.

Professional value is measured as a mediating factor between WPV and SRH using the Professional Value Questionnaire for Medical Staff, developed by Gu et al. in 2015. This tool is based on the Work Value Questionnaire and the Minnesota Satisfaction Questionnaire and comprises 37 items across five dimensions: intrinsic, external, social, altruistic, and leisure values. Intrinsic values focus on motivation derived from the work itself, including personal influence, clarity of goals, responsibilities, feedback, accountability, and interest. External values emphasize material rewards such as salary, social status, wealth, promotion, and compensation. Social values pertain to workplace incentives, such as relationships, recognition, fairness, training, and family support. Altruistic values arise from contributing to society, helping others, and deriving satisfaction from serving others. Finally, leisure values address work-life balance, including autonomy, flexibility, job stability, and a supportive work environment.

To explore the influence of WPV on the health of HCWs, we also controlled for other control variables such as gender, age, education level, and income, as discussed in earlier studies [10, 16, 34]. For detailed variable definitions, please refer to Supplementary Table S1.

### Estimation Models

To investigate the impact of WPV on HCWs’ health, we conducted a regression analysis. Given that the dependent variable is an ordinal variable, an Ordered Logit model was employed in the baseline regression, as shown in Equations 1, 2 below:

$${SRH}_{i}^{*}=\alpha +\beta {WPV}_{i}+\gamma X_{i}^{\prime }+\lambda_{j}+\varepsilon_{i}$$ (1)

$${SRH}_{i}=\left\{\begin{array}{c} 1\;if\;{SRH}_{i}^{*}\leq \mu_{1}\\ 2\;if\;\mu_{1}<{SRH}_{i}^{*}\leq \mu_{2}\\ 3\;if\;\mu_{2}<{SRH}_{i}^{*}\leq \mu_{3}\\ 4\;if\;\mu_{3}<{SRH}_{i\;}^{*}\leq \mu_{4}\\ 5\;if\;\mu_{4}<{SRH}_{i\;}^{*}\leq \mu_{5} \end{array}\right.\;\begin{array}{c} \left(Very\;Unhealthy\right)\\ \left(Unhealthy\right)\\ \left(Average\right)\\ \left(Healthy\right)\\ \left(\text{Very }Healthy\right) \end{array}$$ (2)

Where ${SRH}_{i}^{*}$ is the latent SRH of HCW i , which is mapped to the observed ${SRH}_{i}$ through the cutoff point $\mu_{j}$ that are estimated together with β and satisfied with $\mu_{1}<\mu_{2}<\mu_{3}<\;\mu_{4}<\;\mu_{5}$ . ${WPV}_{i}$ is the explanatory variable that we are interested in, representing the experience of workplace violence by HCW i . $X_{i}^{\prime }$ is a column vector of control variables that may affect HCWs’ SRH , including gender, age, education, income, marriage, working year, night shift, seniority, position, job, department, employment, hospital level, and hospital category. $\lambda_{j}$ is the fixed effect, and $\varepsilon_{i}$ is the residual term.

Additionally, this study aims to investigate the marginal treatment effect (MTE) of WPV, specifically how WPV influences the probability of HCWs’ SRH assuming each value, with other control variables set to their mean. Following the methods of Aakvik, A., J. J. Heckman and E. J. Vytlacil [35] and Huang, B., Y. Lian and W. Li [36], we estimated the MTE of health education on the health of migrants based on the above benchmark model [34].

To ensure more reliable estimates, we employed a multiple linear model to examine the effect of WPV on HCWs’ SRH. While the independent variables are ordered, following the practice in empirical research, the study’s robustness could be damaged if the linear model provides similar results [37]. The model is outlined by Equation 3 below:

$${SRH}_{i}=\alpha +\beta {WPV}_{i}+\gamma X_{i}^{\prime }+\lambda_{j}+\varepsilon_{i}$$ (3)

In this equation, ${SRH}_{i}$ represents the SRH of HCWs i . α is a constant. ${WPV}_{i}$ represents the WPV experience of HCWs i . $X_{i}^{\prime }$ is the same set of control variables as Model [1]. $\lambda_{j}$ is the fixed effect. $\varepsilon_{i}$ is the residual term, and to mitigate the heteroskedasticity problem, we used robust standard errors in the estimation.

To mitigate potential endogeneity, we initially employed propensity score matching (PSM) to minimize the selection bias issues. In practice, the probability that a HCW is subject to WPV is related to their characteristics and thus may lead to selection bias in estimation. This paper corrected the self-selection bias by PSM. By Smith and Todd’s standard [29], we selected the following control variables for the matching process: gender, age, education, marriage, income, work experience, night shifts, seniority, and position. Another potential concern is that our estimates reflect the fact that HCWs with worse health are more likely to suffer from WPV. This potential endogeneity could introduce bias into our estimates, which we address using an instrumental variable (IV). We used the average WPV level from hospitals of the same tier, excluding the HCW’s own hospital, as the IV. This method satisfies the requirement of relevance and exclusion. The WPV level in peer hospitals is strongly associated with the likelihood of WPV exposure for HCWs, as higher WPV levels in these hospitals increase the probability of elevated WPV levels in the worker’s hospital, thereby raising their risk of experiencing WPV. Simultaneously, the WPV levels in other hospitals of the same tier do not directly affect the HCW’s health, satisfying the requirement of exclusion.

## Results

### Descriptive Statistics

Our survey included 25 regional secondary- and tertiary-care hospitals across China. A nationally representative cohort of 4,255 Chinese HCWs was selected. Descriptive statistics are shown in Table 1. Our study revealed that 50.97% of HCWs reported being healthy, a figure significantly lower than the average among Chinese adults, highlighting an important issue that warrants attention [27].

**TABLE 1:** Descriptive statistics (China. 2022–2023).

Variable	Definition	Obs	Mean (%)	S.D.	Min	Max
Explained variable						
SRH	Very unhealthy = 1	55	1.29	0.816	1	5
	Unhealthy = 2	461	10.83			
	Average = 3	1,570	36.90			
	Healthy = 4	1902	44.70			
	Very healthy = 5	267	6.27			
Explanatory variable						
WPV	Workplace violence	4,255	2.189	2.846	0	15
Control variables						
Gender	Female = 0	3,161	74.29	0.437	0	0
	Male = 1	1,094	25.71			
Age	Year	4,255	35.887	8.791	22	60
Education	Year	4,255	15.943	1.429	12	22
Income	CNY	4,255	6,268.376	2,488.098	1,500	11,000
Marriage	No = 0	913	21.46	0.411	0	1
	Yes = 1	3,342	78.54			
Working Year	(0,1] = 1	182	4.28	0.890	1	4
	(1,5] = 2	739	17.37			
	(5,10] = 3	1,168	27.45			
	(10,] = 4	2,166	50.90			
Night Shift	No = 0	1,552	36.47	0.481	0	1
	Yes = 1	2,703	63.53			
Seniority	Not reported = 1	369	8.67	0.930	1	5
	Junior = 2	1741	40.92			
	Intermediate = 3	1,390	32.67			
	Deputy senior = 4	647	15.21			
	Senior = 5	108	2.54			
Position	Intern/student/trainee = 1	70	1.65	0.498	1	4
	Employee = 2	3,225	75.79			
	Administration manager = 3	878	20.63			
	Hospital manager = 4	82	1.93			
Job		4,255	2.256	1.378	1	6
Department		4,255	2.354	1.175	1	4
Employment		4,255	1.555	0.539	1	3
Hospital Level		4,255	2.349	1.687	1	5
Hospital Category		4,255	1.843	0.363	1	2

(1) Education level is a continuous variable, specifically, senior middle school/technical secondary school = 12, junior college = 15, undergraduate = 16, postgraduate = 19, and doctorate = 22. (2) Job is a classified variable, specifically, Physician = 1, Nurse/midwife = 2, Pharmacist = 3, Allied health professional (therapist/radiographer/assistant) = 4, Administrative or clerical worker = 5, Other = 6. (3) Department is a classified variable, specifically, General medicine = 1, General surgery = 2, Medical auxiliary/ancillary = 3, Other = 4. (4) Hospital Level is a classified variable, specifically, Tertiary A = 1, Tertiary B = 2, Secondary A = 3, Secondary B = 4, Others = 5. (5) Hospital category is a classified variable, specifically, Specialty hospital = 1, General hospital = 2.

As for the key variable of interest in this paper, WPV, it has a mean value of 2.189, which means that, on average, HCWs are subjected to one type of WPV. HCWs who experienced WPV were treated as the treatment group, and those who did not were the control group. Supplementary Table S2 shows that 2,454 HCWs (57.67%) reported experiencing WPV at least once in this study. This prevalence is lower than the global average of 78.9% reported in previous research [16]. Furthermore, it could be found that, compared to HCWs who did not suffer WPV, those who experienced WPV have a significantly worse SRH, which is statistically significant at the 1% level. Further investigation is warranted to clarify the causal relationship between workplace violence and SRH outcomes and to identify potential underlying mechanisms.

### Benchmark Regression and Robust Test

Table 2 shows regression analysis reveals that WPV significantly harms Chinese HCWs SRH. Ordered Logit results (Column 1) show a one-unit WPV increase reduces the odds of SRH improvement by 12.9%, with statistical significance (1% level). Marginal effects indicate WPV lowers the probability of “healthy,” “very healthy” SRH by 2.4% and 0.8%, respectively, while raising “very unhealthy” (0.2%), “unhealthy” (1.2%), and “average” (1.7%) probabilities. Multiple linear models (Column 7) confirm WPV’s negative SRH impact, aligning with baseline findings.

**TABLE 2:** The effect of workplace violence on the self-rated health of healthcare workers (China, 2022–2023).

Variables	(1)	(2)	(3)	(4)	(5)	(6)	(7)
	SRH						
	Ordered logit model						OLS model
	Total effect	Marginal effects					
		Very unhealthy	Unhealthy	Average	Healthy	Very healthy	
WPV	0.871***	0.002***	0.012***	0.017***	−0.024***	−0.008***	−0.056***
	(0.011)	(0.000)	(0.001)	(0.002)	(0.002)	(0.001)	(0.005)
Gender	1.237**	−0.003**	−0.019**	−0.027**	0.036**	0.012**	0.071**
	(0.103)	(0.001)	(0.007)	(0.011)	(0.014)	(0.005)	(0.033)
Age	0.998	0.000	0.000	0.000	−0.000	−0.000	−0.001
	(0.005)	(0.000)	(0.000)	(0.001)	(0.001)	(0.000)	(0.002)
Education	0.983	0.000	0.002	0.002	−0.003	−0.001	−0.009
	(0.023)	(0.000)	(0.002)	(0.003)	(0.004)	(0.001)	(0.010)
Income	1.000	0.000	0.000	0.000	−0.000	−0.000	0.000
	(0.000)	(0.000)	(0.000)	(0.000)	(0.000)	(0.000)	(0.000)
Marriage	1.185^**^	−0.002^*^	−0.015^*^	−0.022^**^	0.029^**^	0.010^*^	0.070**
	(0.103)	(0.001)	(0.008)	(0.011)	(0.015)	(0.005)	(0.035)
Working Year	0.837***	0.002***	0.016***	0.023***	−0.030***	−0.010***	−0.072***
	(0.045)	(0.001)	(0.005)	(0.007)	(0.009)	(0.003)	(0.022)
Night Shift	0.645***	0.006***	0.039***	0.055***	−0.075***	−0.025***	−0.178***
	(0.044)	(0.001)	(0.006)	(0.008)	(0.011)	(0.004)	(0.027)
Seniority	1.055	−0.001	−0.005	−0.007	0.009	0.003	0.022
	(0.056)	(0.001)	(0.005)	(0.007)	(0.009)	(0.003)	(0.022)
Position	1.409***	−0.004***	−0.030***	−0.043***	0.059***	0.020***	0.138***
	(0.105)	(0.001)	(0.007)	(0.009)	(0.013)	(0.004)	(0.030)
Cons	-						3.639***
							(0.178)
Fixed Effect	Yes	Yes	Yes	Yes	Yes	Yes	Yes
Observations	4,255	4,255	4,255	4,255	4,255	4,255	4,255
(Pseudo) R^2^	0.046	0.046	0.046	0.046	0.046	0.046	0.104

(1) *, **, and *** represent significance at the 10%, 5%, and 1% levels, respectively. (2) The numbers in parentheses are robust standard errors. (3) The coefficients in column 1 are presented as odds ratio.

### Endogeneity Solving

The study confirms that WPV significantly harms HCWs’ SRH. To strengthen reliability and address potential endogeneity, the analysis employed two methods: PSM and IV. First, PSM was applied using a 1:1 nearest-neighbor approach (caliper = 0.01). Supplementary Table S3 and Supplementary Figure S1 confirmed balance between treatment and control groups post-matching, with no significant differences (p > 0.1 for all covariates). Kernel density plots (Supplementary Figure S1) demonstrated aligned distributions, validating the common support assumption. Table 3 (columns 1–7) showed WPV’s negative coefficients remained statistically significant, reinforcing baseline results and confirming WPV’s adverse health impact after correcting for selection bias.

**TABLE 3:** Regression results after mitigating endogeneity (China, 2022–2023).

Variables	(1)	(2)	(3)	(4)	(5)	(6)	(7)	(8)
	SRH							
	PSM							IV
	Ordered Logit Model						OLS Model	
	Total Effects	Marginal Effects						
		Very Unhealthy	Unhealthy	Average	Healthy	Very Healthy		
Violence	0.862***	0.002***	0.012***	0.020***	−0.024***	−0.010***	−0.060***	−0.053***
	(0.016)	(0.000)	(0.002)	(0.002)	(0.003)	(0.001)	(0.007)	(0.007)
Gender	1.197	−0.002	−0.014	−0.025	0.029	0.012	0.058	0.069^**^
	(0.140)	(0.002)	(0.009)	(0.016)	(0.019)	(0.008)	(0.047)	(0.033)
Age	1.000	−0.000	−0.000	−0.000	0.000	0.000	0.000	−0.001
	(0.007)	(0.000)	(0.001)	(0.001)	(0.001)	(0.000)	(0.003)	(0.002)
Education	0.963	0.000	0.003	0.005	−0.006	−0.002	−0.015	−0.009
	(0.031)	(0.000)	(0.003)	(0.004)	(0.005)	(0.002)	(0.013)	(0.010)
Income	1.000	0.000	0.000	0.000	−0.000	−0.000	−0.000	0.000
	(0.000)	(0.000)	(0.000)	(0.000)	(0.000)	(0.000)	(0.000)	(0.000)
Marriage	1.243*	−0.003*	−0.017*	−0.030*	0.035*	0.015*	0.090*	0.070**
	(0.151)	(0.002)	(0.010)	(0.017)	(0.020)	(0.008)	(0.050)	(0.035)
Working Year	0.860*	0.002*	0.012*	0.021*	−0.024*	−0.010*	−0.060*	−0.073***
	(0.067)	(0.001)	(0.006)	(0.011)	(0.013)	(0.005)	(0.032)	(0.022)
Night Shift	0.673***	0.005***	0.031***	0.055***	−0.065***	−0.027***	−0.160***	−0.180***
	(0.063)	(0.002)	(0.008)	(0.013)	(0.015)	(0.006)	(0.038)	(0.027)
Seniority	0.989	0.000	0.001	0.002	−0.002	−0.001	−0.008	0.021
	(0.076)	(0.001)	(0.006)	(0.011)	(0.012)	(0.005)	(0.031)	(0.022)
Position	1.398***	−0.004***	−0.026***	−0.046***	0.055***	0.022***	0.127***	0.138***
	(0.143)	(0.002)	(0.008)	(0.014)	(0.017)	(0.007)	(0.042)	(0.030)
Cons	-						3.639***	3.318***
							(0.178)	(0.198)
Fixed Effect	Yes	Yes	Yes	Yes	Yes	Yes	Yes	Yes
Observations	2,208	2,208	2,208	2,208	2,208	2,208	2,208	4,255
(Pseudo) R^2^	0.040	0.040	0.040	0.040	0.040	0.040	0.091	0.104

Note: (1) *, **, and *** represent significance at the 10%, 5%, and 1% levels, respectively. (2) The numbers in parentheses are robust standard errors. (3) The coefficients in Column 1 are presented as odds ratio.

Second, the IV approach used the average WPV level from peer hospitals (same tier, excluding the respondent’s hospital) as the instrument. IV regression results (Table 3, column 8) confirmed instrument validity: the unidentifiable test (p = 0.000) proved strong correlation, while the Kleibergen-Paap Wald F-statistic (65.590) exceeded the threshold (F > 10), ruling out weak instrument concerns. The IV estimate for WPV (−0.053, p < 0.01) further validated its negative health effect. Together, PSM and IV analyses robustly support the conclusion that WPV deteriorates HCWs’ health, with consistent findings across methodologies.

### Further Analysis on the Type of WPV

We also investigate the negative impact of each category type of WPV on the health of HCWs. WPV can be categorized into 5 types [16], and Table 4 provides estimates of health shocks for all WPV types. All types of WPV have a statistically significant negative impact on the SRH of HCWs, with physical violence being the most destructive. Specifically, for each unit increase in physical violence, the probability that a HCW’s SRH would increase by one level would decrease by 30.2%.

**TABLE 4:** Effects of different workplace violence on the self-rated health of healthcare workers (China, 2022–2023).

Variables	(1)	(2)	(3)	(4)	(5)
	SRH				
	Ordered logit model				
Physical Violence	0.698***				
	(0.031)				
Psychological Abuse		0.712***			
		(0.019)			
Verbal Threats			0.700***		
			(0.026)		
Verbal Sexual Harassment				0.759***	
				(0.042)	
Physical Sexual Harassment					0.739***
					(0.060)
Controls	Yes	Yes	Yes	Yes	Yes
Fixed Effect	Yes	Yes	Yes	Yes	Yes
Observations	4,255	4,255	4,255	4,255	4,255
Pseudo R^2^	0.037	0.046	0.040	0.033	0.032

Note: (1) *, **, and *** represent significance at the 10%, 5%, and 1% levels, respectively. (2) The numbers in parentheses are robust standard errors. (3) The coefficients are presented as odds ratio.

### Mechanism Analysis

The study demonstrates that WPV significantly impairs HCWs’ SRH by eroding their professional values. Panel A of Table 5 shows WPV negatively impacts both overall professional values and all five sub-dimensions (p < 0.01). Each unit increase in WPV reduces the likelihood of improved total professional values by 15.1%, with particularly strong effects on leisure values, suggesting severe disruptions to work-life balance. The consistent negative effects across all dimensions underscore WPV’s pervasive harm to HCWs’ professional identity and motivation. Panel B reveals professional values positively influence SRH, with altruistic values showing the strongest effect (34.3% increased odds of better SRH per unit increase). External (17.6%) and internal (15.7%) values also significantly boost SRH, while societal (11.3%), leisure (11.1%), and total scores (3.9%) show smaller but meaningful effects. These results highlight those professional values, especially altruism, serve as key protective factors for HCWs’ health perceptions. Together, these findings establish professional value erosion as a critical mechanism linking WPV to poorer SRH. The study suggests interventions should both prevent WPV and strengthen professional values, particularly altruism and work-life balance, to safeguard HCWs’ health. The robust, multi-dimensional evidence supports comprehensive policy approaches addressing both violence reduction and value reinforcement.

**TABLE 5:** The mediating effect of professional value (China, 2022–2023).

Panel A						
Variables	(1)	(2)	(3)	(4)	(5)	(6)
	Ordered Logit Model					
	Total	Inside	Outside	Society	Altruistic	Leisure
Violence	0.849***	0.909***	0.865***	0.867***	0.872***	0.830***
	(0.010)	(0.010)	(0.010)	(0.010)	(0.010)	(0.011)
Controls	Yes	Yes	Yes	Yes	Yes	Yes
Fixed Effect	Yes	Yes	Yes	Yes	Yes	Yes
Observations	4,255	4,255	4,255	4,255	4,255	4,255
Pseudo R2	0.015	0.012	0.021	0.017	0.019	0.023

Panel B						
	(1)	(2)	(3)	(4)	(5)	(6)
	Ordered Logit Model					
	SRH	SRH	SRH	SRH	SRH	SRH
Total	1.039***					
	(0.002)					
Inside		1.157***				
		(0.011)				
Outside			1.176***			
			(0.010)			
Society				1.113***		
				(0.006)		
Altruistic					1.343***	
					(0.023)	
Leisure						1.111***
						(0.006)
Controls	Yes	Yes	Yes	Yes	Yes	Yes
Fixed Effect	Yes	Yes	Yes	Yes	Yes	Yes
Observations	4,255	4,255	4,255	4,255	4,255	4,255
Pseudo R^2^	0.088	0.065	0.080	0.080	0.068	0.077

Note: (1) *, **, and *** represent significance at the 10%, 5%, and 1% levels, respectively. (2) The numbers in parentheses are robust standard errors. (3) Professional value has 5 subscore, including inside value, extrinsic value, society value, altruistic value, and leisure value. (4) The coefficients are presented as odds ratio.

### Heterogeneity Analysis

The study examines how WPV differentially affects HCWs’ health across demographic groups (Table 6). While WPV harms all HCWs, effects vary significantly: (i) Gender: Females show greater SRH deterioration (85.6% of original improvement probability) than males (89.8%), showing a 4.2% gap. (ii) Age: Younger HCWs experience more severe health impacts than older colleagues. (iii) Seniority: Junior staff (85.1% probability) face 3.5% greater SRH reduction than senior workers (88.6%), reflecting experience/socioeconomic buffers. (iv) Hospital level: Non-tertiary hospital workers (85.5%) show 3.3% worse outcomes than tertiary hospital staff (88.8%), indicating resource disparities.

**TABLE 6:** Heterogeneous effects of workplace violence on the self-rated health of healthcare workers (China, 2022–2023).

Variables	(1)	(2)	(3)	(4)	(5)	(6)	(7)	(8)
	Self-rated health							
	Ordered logit model							
	Gender		Age		Seniority		Hospital level (3A)	
	Female	Male	Low	High	Low	High	Non	Yes
Violence	0.856^***^	0.898^***^	0.854^***^	0.885^***^	0.851^***^	0.886^***^	0.855^***^	0.888^***^
	(0.013)	(0.019)	(0.016)	(0.014)	(0.016)	(0.015)	(0.015)	(0.016)
Controls	Yes	Yes	Yes	Yes	Yes	Yes	Yes	Yes
Fixed Effect	Yes	Yes	Yes	Yes	Yes	Yes	Yes	Yes
Observations	3,161	1,094	2,188	2067	2,110	2,145	2,312	1943
Pseudo R^2^	0.051	0.048	0.049	0.050	0.044	0.050	0.049	0.040
Empirical P value	0.038**		0.067*		0.047**		0.064*	

Note: (1) *, **, and *** represent significance at the 10%, 5%, and 1% levels, respectively. (2) The numbers in parentheses are robust standard errors. (3) The coefficients are presented as odds ratio. (4) Age is grouped based on the sample median of 35 years, with individuals aged 35 or younger classified as “Low” and those older than 35 categorized as “High.” Seniority is divided by professional experience: junior-level and lower positions are classified as “Low,” while intermediate, associate senior, and senior roles are classified as “High” Hospital level is categorized based on whether the hospital is tertiary or non-tertiary: tertiary hospitals are marked as “Yes,” and non-tertiary as “No.” (5) The “Empirical P-value” is obtained by Fisher’s Permutation test and used to test the significance of the difference in the coefficients of WPV within groups.

These findings demonstrate WPV’s universal harm while revealing critical vulnerabilities: Female HCWs experience disproportionately severe effects; Less experienced/younger workers show greater susceptibility; Resource-constrained settings exacerbate impacts. And the results underscore the need for targeted interventions addressing these differential vulnerabilities through gender-sensitive protections, enhanced support for junior staff, and resource allocation to non-tertiary hospitals.

## Discussion

### Main Findings

Healthcare workers are a high-risk group for exposure to workplace violence, and such adverse experiences exacerbate their physical and psychological vulnerabilities, posing a serious threat to the normal functioning of the healthcare system. However, limited empirical evidence exists on the extent to which workplace violence undermines the health of healthcare workers, and little is known about the underlying mechanisms and heterogeneous effects. These gaps constrain the development and implementation of effective policies. To address this issue, this study employs cross-sectional survey data from 4,255 healthcare workers in China to investigate these questions, thereby contributing to the literature on workplace violence and informing the design of relevant intervention policies.

Our study finds that WPV significantly impairs HCWs’ health, a finding that remains reliable under various robustness tests and is an empirical addition to research on HCWs’ health influencing factors [16]. Given the healthcare’s challenging WPV environment [3, 17], this finding has important practical implications. Therefore, we need to emphasize the health of HCWs and take adequate measures to stop the occurrence. Our study suggests that WPV undermines the SRH of HCWs by diminishing their professional values and, in turn, their SRH. A study involving nursing students found a significant negative correlation between WPV and professional identity [38], and another study by Zhang et al. [36] found that WPV affects the sleep quality of psychiatric nurses through professional identity. There is a complex interaction between professional values and health. Conflicts between professional values and reality can lead to psychological stress for HCWs. For instance, the values of medical staff conflict with the reality of their work can lead to guilt, anxiety, and burnout, thus affecting their mental health via a path like conflicting values lead to the accumulation of internal stress, and internal stress leads to impaired mental health (e.g., anxiety, depression). The altruistic value of medical professionals may be taken to extremes, leading to over-commitment to their work at the expense of their own health. The possible path is that altruistic tendencies cause neglect of rest and self-care, which leads to physical fatigue and decreased immunity [39].

Our study concludes that physical violence exhibits the most pronounced effect, followed by oral threats, psychological abuse, physical sexual harassment, and verbal sexual harassment. As mentioned in a few studies [40], different types of WPV might affect health among medical staff. However, to our knowledge, no study has attempted to quantify this differential effect. The present study makes a unique contribution by quantifying the adverse impact of each type of WPV, adding new empirical evidence to the existing body of studies. Possible reasons for our findings are that physical violence is the most direct and immediate threat to safety, and bodily injury can lead to long-term health issues like chronic pain. Plus, the visibility of physical violence might make it more likely to be reported, which could affect study results [41].

The findings of heterogeneity indicate that the female gender is associated with an elevated risk of WPV-related health damage. This observation is consistent with previous studies [42]. First, women are overrepresented in high-risk sectors, including nursing and midwifery, while male workers predominate among physicians, dentists, and pharmacists. In this case, we can see a clear power imbalance in client-facing roles [43]. Second, biological vulnerability due to heightened stress among women is severe. Female nurses show 23% higher PTSD rates post-WPV than males [44]. Another possibility is the underreporting in male-dominated fields since most males did not like to disclose.

Furthermore, younger HCWs and those with lower seniority are more vulnerable, and this finding aligns with previous studies [9]. Possible explanations are that younger, lower-graded HCWs are less able to withstand adverse external shocks, i.e., WPV, and that, in the Chinese social context, these HCWs lack adequate social skills to cope with WPV and its negative impacts. WPV has a more severe health deterioration effect on HCWs in non-tertiary hospitals. Previous studies indicated that more than half of the medical WPV events occurred in tertiary hospitals due to the higher volume of patients and higher expectations of the patients. However, the present study reveals that HCWs in non-tertiary hospitals were experiencing more critical health situations that necessitate heightened attention in future research. The underlying reasons for this discrepancy are not fully elucidated, however, it is hypothesized that tertiary hospitals are equipped with more resources, which could be used to relieve the effect of WPV after such events and tertiary hospital managers paid more attention to the measures to prevent or tackle WPVs, and this finding requires more attention in future research [45–47].

### Policy Implications

This study highlights key policy measures to address WPV against HCWs. For HCWs, it emphasizes proactive steps to protect their health, rights, and working conditions. Policymakers must prioritize reducing WPV by strengthening institutional frameworks tailored to each country’s socio-economic context, such as stricter penalties to deter perpetrators. Enhancing HCWs’ professional values through recognition programs, career development, and public appreciation can mitigate WPV’s health impacts. The study also calls for targeted interventions for vulnerable groups, including females, younger HCWs, and lower-grade HCWs, by ensuring equitable access to anonymous reporting and decision-making channels. Non-tertiary hospitals, often under-resourced, require exceptional support to address systemic challenges. Combining legal, professional, and equity-focused strategies, a multifaceted approach is essential to create safer healthcare environments.

### Limitations

First, although various methods were employed to enhance the reliability of the results, this study is based on cross-sectional data due to data limitations. Second, the data was collected in China, as the situation differs from one country to another, incorporating data from other nations could enhance the generalizability of the findings. Finally, this study focused solely on the mechanism of professional values, and future research could contribute by exploring additional mechanisms to provide a more comprehensive understanding of this relationship.

### Conclusion

This study confirms that WPV severely harms HCWs’ health, with physical violence being most detrimental. Female, younger, lower-ranking, and non-tertiary hospital staff face higher risks. Professional values partially mediate this harm. These findings call for urgent WPV prevention policies and targeted support for vulnerable groups. Future research should prioritize interventions to safeguard HCWs’ wellbeing.

## Data Availability

The data used to support the findings of this study are available from the corresponding authors upon request.
