# Supplementary material for: The Effect of Workplace Violence on the Health of Healthcare Workers: Empirical Evidence From a Multicenter Cross-Sectional Study in China
Source: Int J Public Health. 2025 Oct 27;70:1608523. doi: 10.3389/ijph.2025.1608523 (PMC12597837; doi:10.3389/ijph.2025.1608523)

**Table S1:** **Description of variables (China, 2022–2023).**

| **Variable** | **Notation** | **Description** |
| --- | --- | --- |
| Self-rated health | *SRH* | 1 = Very unhealthy, 2 = Unhealthy,  3 = Average, 4 = Healthy, 5 = Very healthy |
| Workplace violence | *WPV* | From 0 to 15 |
| Gender | *Gender* | 0 = female, 1 = male |
| Age | *Age* | Unit: year |
| Education | *Education* | Unit: year |
| Monthly income | *Income* | Unit: CNY |
| Marital status | *Marriage* | 0 = unmarried, 1 = married |
| Working Year | *Working Year* | 1 = (0,1], 2 = (1,5], 3 = (5,10], 4 = (10,] |
| Night Shift | *Night Shift* | 0 = no, 1 = yes |
| Seniority | *Seniority* | 1 = Not reported, 2 = Junior, 3 = Intermediate, 4 = Deputy senior, 5 = Senior |
| Position | *Position* | 1 = Intern/student/trainee, 2 = Employee, 3 = Administration manager, 4 = Hospital manager |

**Table S2: The gap in self-rated health between the healthcare workers with and without workplace violence (China, 2022–2023).**

| Variable | WPV | | | | T-test |
| --- | --- | --- | --- | --- | --- |
|  | No (N=1801)  Control Group | | Yes (N=2454)  Treatment Group | | Diff |
|  | Mean | S.D. | Mean | S.D. | (Yes-No) |
| SRH | 3.637 | 0.762 | 3.292 | 0.824 | -0.345*** |

Note: *, **, and *** represent significance at the 10%, 5%, and 1% levels, respectively.

**Table** **S3: The results of the balance test of the propensity score matching method (China, 2022–2023).**

| Variable | Matching | Mean | | | %reduct  \|bias\| | T-test | |
| --- | --- | --- | --- | --- | --- | --- | --- |
|  |  | Treated | Control | %Bias |  | t | P > t |
| Gender | U | 0.270 | 0.239 | 7.10 |  | 2.280 | 0.023 |
|  | M | 0.270 | 0.281 | -2.60 | 63.00 | -0.890 | 0.371 |
| Education | U | 16.05 | 15.80 | 16.90 |  | 5.450 | 0.000 |
|  | M | 16.04 | 16.05 | -0.60 | 96.60 | -0.200 | 0.845 |
| Income | U | 6510 | 5940 | 23.00 |  | 7.430 | 0.000 |
|  | M | 6508 | 6603 | -3.90 | 83.20 | -1.360 | 0.173 |
| Marriage | U | 0.798 | 0.768 | 7.40 |  | 2.390 | 0.017 |
|  | M | 0.798 | 0.800 | -0.50 | 93.30 | -0.180 | 0.859 |
| Night Shift | U | 0.689 | 0.562 | 26.30 |  | 8.520 | 0.000 |
|  | M | 0.689 | 0.675 | 2.80 | 89.30 | 1.010 | 0.312 |
| Position | U | 2.246 | 2.204 | 8.40 |  | 2.710 | 0.007 |
|  | M | 2.246 | 2.250 | -0.90 | 89.30 | -0.310 | 0.758 |

**Figure S1. Kernel density of the treatment and control groups (China, 2022–2023).**


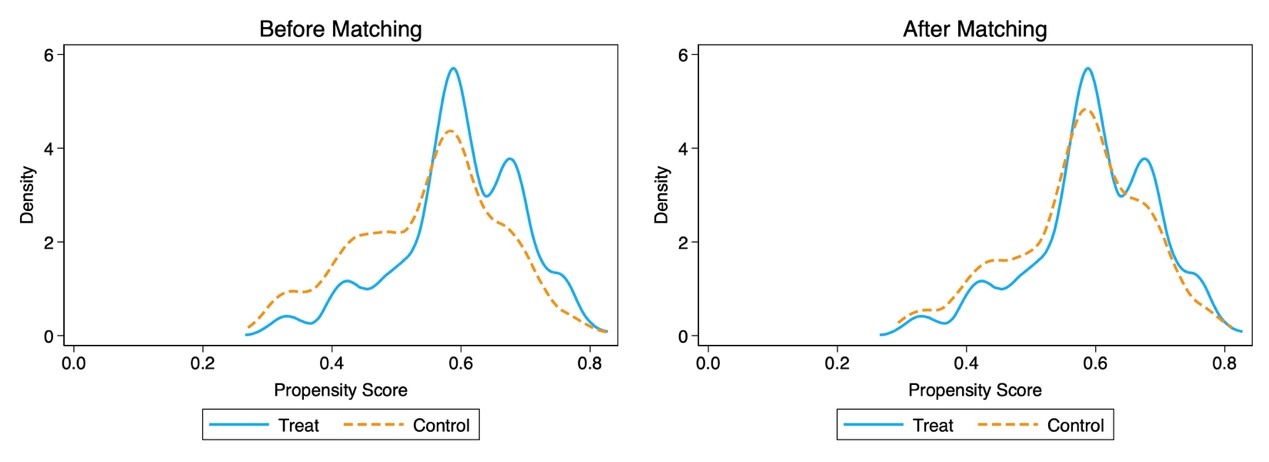

Supplement: Supplementary file 1 [file Supplementaryfile1.docx]
